# Bullying the Brain? Longitudinal Links Between Childhood Peer Victimization, Cortisol, and Adolescent Brain Structure

**DOI:** 10.3389/fpsyg.2018.02706

**Published:** 2019-01-11

**Authors:** Mieke R. du Plessis, Sanny Smeekens, Antonius H. N. Cillessen, Sarah Whittle, Berna Güroǧlu

**Affiliations:** ^1^Institute of Psychology, Leiden University, Leiden, Netherlands; ^2^Department of Psychology, Open University, Heerlen, Netherlands; ^3^Behavioural Science Institute, Radboud University, Nijmegen, Netherlands; ^4^Melbourne Neuropsychiatry Centre, Department of Psychiatry, The University of Melbourne and Melbourne Health, Melbourne, VIC, Australia

**Keywords:** victimization, cortisol, stress, ventrolateral prefrontal cortex, stress, brain structure

## Abstract

**Background:** Childhood peer victimization is a stressful life experience associated with long-lasting adverse psychological consequences. While there is some evidence that victimization is associated with alterations in brain function, little is known about effects on brain structure. This study explored the relationships between childhood peer victimization, cortisol, and adolescent ventrolateral prefrontal cortex (vlPFC) structure in a sample of healthy children.

**Methods:** A total of 50 (*M*_age_ = 9.29 years at baseline) children participated in this longitudinal study. We examined whether diurnal cortisol levels (assessed at baseline) moderated the link between children’s self-reported peer victimization (assessed at baseline) and vlPFC surface area, gray matter volume, and thickness 5 years later.

**Results:** For boys, cortisol levels moderated the association between victimization and brain structure. For boys with a low daily cortisol output (assessed as area under the curve; AUC), high victimization was associated with a smaller right vlPFC surface area, and for boys with a high AUC, high victimization was associated with a larger right vlPFC surface area. In addition, for boys with a steeper diurnal slope, high victimization was associated with a smaller right vlPFC surface area, and for boys with a low flatter diurnal slope, high victimization was associated with a larger right vlPFC surface area.

**Conclusion:** These results indicate the differential influence of cortisol on the relationship between victimization and brain structure. Findings suggest that victimization may have differential effects on brain development in boys who are more versus less biologically sensitive to stress.

## Introduction

Peer victimization is characterized as being the habitual target of peers’ physical or emotional aggression (Olweus, 1993). Peer victimization is a global issue affecting around 30% of children in any given month (United Nations Children’s Fund, 2007) and for about 11% of children, this abuse occurs on a regular basis. Peer victimization is often a stable construct where children who are victimized at one point in time tend to be victimized throughout their childhood and adolescence (Kumpalainen et al., 1999). Sex is a robust correlate of bullying with boys both at greater risk of perpetrating and experiencing peer victimization than girls (e.g., Nansel et al., 2001).

There is mounting evidence that peer victimization is an experience that can have long-lasting adverse psychological consequences. For example, being victimized during school years has been shown to be detrimental to academic functioning, social relationships, self-perception, cognition, physical health, and mental health (e.g., Austin and Joseph, 1996; Grills and Ollendick, 2002; Bellmore and Cillessen, 2006; Esbensen and Carson, 2009; Juvonen et al., 2011). Peer victimization has also been associated with altered neurobiology, and indeed, it has been suggested that this is one mechanism by which victimization can impact psychological functioning (Rudolph et al., 2016), with the ventrolateral prefrontal cortex (vlPFC) being particularly implicated. For example, functional neuroimaging studies show that social exclusion is associated with activation in the vlPFC, and vlPFC activation correlates negatively with social distress during exclusion (Eisenberger et al., 2003; Masten et al., 2009; Yanagisawa et al., 2011). Stimulation of the vlPFC with transcranial direct stimulation during social exclusion dampens distress (Riva et al., 2015). Children who are habitually excluded by their peers show more lateral prefrontal cortex (lPFC) activity in the face of social stress (Will et al., 2016) and when resisting risky behaviors (Telzer et al., 2017), and show greater vlPFC activation when receiving negative social feedback than do children who are not peer-rejected (Lee et al., 2014).

While the findings linking peer victimization to altered neurobiology are intriguing, there are two critical gaps in the literature. First, the majority of relevant studies have focused on brain function. Presently the associations between victimization and brain *structure* remain obscure. Investigation of PFC structure is of interest given that many of the psychological consequences of peer victimization have been associated with structural alterations in the PFC. For example, reductions in PFC surface area have been found in adolescents with depression (Schmaal et al., 2017), and reduced vlPFC volumes have been found in anxiety disorder patients (Shang et al., 2014). The experience of other types of environmental adversity has been associated with reduced vlPFC thickness in adolescents (Gold et al., 2016).

Second, neurobiological research has not taken into account the fact that not all children exposed to peer victimization experience adverse consequences. To understand the nature of differential response to peer victimization, individual differences in vulnerability or sensitivity factors need to be investigated. In this regard, individual differences in the activity of the hypothalamic-pituitary-adrenal (HPA) axis may account for variations in the effects of peer victimization.

The hypothalamic-pituitary-adrenal-axis (HPA-axis; Hansen et al., 2006; Vaillancourt et al., 2008) regulates the secretion of cortisol. The basal secretion of cortisol shows a stable diurnal pattern. In general, the diurnal cortisol curve shows increases prior to awakening and reaches its peak 30 min after waking. This increase is known as the cortisol awakening response (CAR: e.g., Fries et al., 2009). During the rest of the day cortisol levels gradually decrease (Hostinar and Gunnar, 2013). The CAR has been suggested as a measure of acute HPA-reactivity distinct from cortisol output throughout the rest of the day (Pruessner et al., 2007). Diurnal slope, on the other hand, has been suggested as a tonic reflection of stress experienced throughout the day. It is also possible to characterize the entire cortisol output across the day by calculating the area under the curve (AUC) for all cortisol measurements on a given day (Pruessner et al., 2007). Abnormal patterns of cortisol secretion during the day (e.g., Fries et al., 2009) as well as in response to stress are associated with physical problems (Miller et al., 2007) and negative outcomes such as problem behavior (e.g., McBurnett et al., 2000; Alink et al., 2008) and psychopathology (Buitelaar, 2013).

It has been posited that adolescence is an important developmental period in which the effects of earlier exposures to stress become evident where individual differences in HPA reactivity might play an important role (Lupien et al., 2009). There is some evidence to suggest that cortisol levels may moderate the effect of peer victimization on outcomes. For example, victimization has been shown to be associated with elevated depressive symptoms only in those individuals with elevated basal cortisol levels (Brendgen et al., 2017), and elevated cortisol response to social challenge (Rudolph et al., 2011). These findings suggest that individuals with hyper-sensitive stress response systems may be less able to cope effectively with peer victimization. While similar research has not been performed regarding neurobiological outcomes, individual differences in HPA-axis function are likely to impact adolescent brain structure given evidence for an effect of glucocorticoid exposure on PFC structure in rodents (Wellman, 2001), and of associations between cortisol levels and PFC volumes in humans (Carrion et al., 2010).

To better understand the role of the HPA-axis on the consequences of bullying, the current study examined the moderating role of HPA-axis function in the link between bullying and brain structure in adolescents. We focused on individual differences in the CAR, diurnal slope, and AUC, and on vlPFC structure specifically. We expected to find a relationship between victimization and the structure of the vlPFC in a way that was dependent on individual differences in CAR, diurnal slope and AUC. Specifically, we hypothesized that victimized children with indices of HPA-axis hyperactivity/hyper-reactivity (i.e., elevated CAR, AUC and flattened slope) would have reduced vlPFC structure relative to those with higher cortisol levels. Finally, we took sex into account, given (a) sex differences in peer victimization, (b) sex differences in neural development (Raznahan et al., 2010) and in function of the vlPFC (Vijayakumar et al., 2014), (c) sex differences n basal cortisol secretion (Schiefelbein and Susman, 2006), and (d) evidence for differential effects of victimization on both cortisol and brain function for boys and girls (Stroud et al., 2002).

## Materials and Methods

### Participants

The sample described in the current study was recruited as part of the Nijmegen Longitudinal Study (NLS), a large ongoing longitudinal study on infant and childhood development, conducted in Nijmegen, Netherlands. The study started in 1998 with a community sample of 129 15-month-old children (van Bakel and Riksen-Walraven, 2002). Families were recruited on the basis of records from local health-care centers in the city of Nijmegen, Netherlands and no specific exclusion criteria were employed. All families with a 15-month-old baby (i.e., 639 families) living in districts with many young families from various socioeconomic backgrounds were contacted. Of the 174 families who responded to an invitation to participate, 129 families were randomly selected (the maximum possible given the resources available for the project). Participants were assessed at 10 time points at ages 15 and 28 months, 5, 7, 9, 12, 13, 14, 16, and 17 years old. The current study examined the links between victimization and cortisol assessed at age 9 (Wave 5) and brain structure assessed at age 14 (Wave 8). Fifty children had brain structure measures at Wave 8 and thus comprised the final sample. The sample included five children whose parents were divorced, measured when the children were 10 years old. The mean level of education of the children’s mothers, on a scale of 1 (elementary school) to 7 (college degree or more), was 5.14 (*SD* = 1.57), while for fathers this was 5.14 (*SD* = 1.73). Consent to participate in the study was obtained from both the child and at least one parent at all time points. The local ethics committee (CMO region Arnhem-Nijmegen, Netherlands) approved this study.

### Procedure and Measures

#### Wave 5 Data Collection

At Wave 5 of the NLS, participants were 9 years old (*M_age_* = 9.29 years, *SD* = 0.37; 50% boys). During a school visit, 118 children completed several questionnaires in a classroom setting, among which a questionnaire assessing peer victimization. Saliva samples were collected from the participants within 4 weeks after the school visit.

#### Wave 5: Self-Reported Victimization

Participants completed the self-report Olweus Bully/Victim questionnaire (Olweus, 1986) at school during a 30–60 min classroom session. The Olweus Bully/Victim questionnaire uses four items to assess whether children are being bullied, with bullying defined as having the following characteristics: “the intention to harm the victim, the repetitive nature of bullying, and the imbalance in power between the victim and the perpetrator(s)” (Solberg and Olweus, 2003, pp 246). Students were asked to indicate on a 5-point scale the extent to which they had been victimized at school during the past school year. The scale ranged from 1 “never,” 2 “once or a few times,” 3 “one or two times a month,” 4 “one or two times a week,” to 5 “three or more times a week.” The following four questions were asked: (1) How many times in this school year has a classmate said mean things about you at school? (2) How many times this year has a classmate hit, kick, or pushed you? (3) How many times this year has a classmate shouted at you? (4) How many times this school year have your classmates bullied you? The four items were summed to yield a total victimization score, with higher scores reflecting higher self-reported victimization (Cronbach’s α = 0.728).

#### Wave 5: Cortisol

Saliva samples were obtained on two consecutive school days (Monday and Tuesday). On each day five samples were taken: two at home in the morning (directly after awakening and 30 min later), two at school (at noon before lunch and in the afternoon before going home), and one at home before bedtime. All involved families agreed to the saliva collection procedure and 97 of 118 children completed the salivary samples at home and at school successfully. Participants with unsuccessful salivary cortisol samples were excluded because of using potentially interfering medication (for attention deficit hyperactivity disorder, allergies, asthma, or diabetes), feeling ill during sampling, or returning sampling packages that may have been thawed too long. Salivary samples were sent to the Biochemisches Labor at the University of Trier, Germany, for analysis using time-resolved fluorescence immunoassay (DELFIA; Dressendörfer et al., 1992). Each child’s samples were analyzed in one assay batch to minimize variability, and duplicate assays were performed to guarantee validity of analysis. If control samples showed cortisol values outside a defined range (±2 *SD*) the whole batch was reanalyzed. The intra-assay coefficients of variation were between 7.1 and 9%. All values >50 nmol/L were considered out of range and were assigned a value 2 *SD* above the mean, as suggested by Kertes and Gunnar (2004).

Of the 50 children who had brain structure measures at Wave 8, 41 had cortisol data at Wave 5. To estimate cortisol awakening response and the diurnal change in cortisol levels, data from both data collection days were averaged after calculating the CAR, diurnal slope, and area under the curve with respect to ground (AUC_ground_). When relating individual characteristics to cortisol levels, averaging cortisol data across days is recommended (Pruessner et al., 2007). Further, it is of note that there was moderate to high stability in cortisol values in our sample across the 2 days (see below), and as such, averaging was thought to increase the reliability of the measures.

The cortisol awakening response (CAR) was calculated as the difference between the morning cortisol measurement (time 2) and the awakening measurement (time 1). The correlation of CAR between Day 1 and Day 2 was 0.60 (*p* < 0.001). The CAR was uncorrelated with the noon, afternoon, and bedtime cortisol measures. The diurnal slope was taken as the difference between the morning cortisol measurement (time 2) and the bedtime measurement (time 5). The correlation of the diurnal slope on Day 1 and Day 2 was 0.47 (*p* < 0.01). Finally, to assess children’s daily cortisol secretion AUC_ground_ was calculated using all five cortisol measurements (see Pruessner et al., 2003). One child did not provide a cortisol sample on the second day for time point three and, as a result, no AUC_ground_ was calculated for them. The correlation of AUC_ground_ between day 1 and day 2 was 0.98 (*p* < 0.001). This correlation remained significant after removal of one outlier.

#### Wave 8 Data Collection

At Wave 8 of the NLS, participants were 14 years old (*M_age_* = 14.64 years, *SD* = 0.18). This wave included a neuroimaging study where participants took part in an fMRI experiment. Of the 118 participants who participated at Wave 5, 83 participants took part in Wave 8, which involved an approximately 2.5 h protocol that also included tasks that are reported elsewhere (Niermann et al., 2015; Tyborowska et al., 2016). Of the 83 participants, 53 who indicated no MRI contraindications (no braces, free of current psychiatric or neurological impairments, no metal implants, and right-handed) participated in the MRI session. Prior to scanning, participants were familiarized with the scanning environment with a mock scanner. The total scanning time was 50 min. Children and their parents received a financial reimbursement for their participation.

### Wave 8: Neuroimaging

#### MRI Acquisition

The MRI data were acquired on a Siemens 3 tesla MAGNETOM Tri MRI scanner (Siemens Medical Solutions) using a 32-channel coil. Structural T1 images were acquired using an MPRAGE sequence (TR = 2300 ms; TE = 3.03 ms; 192 sagittal slices; 1.0 mm × 1.0 mm × 1.0 mm voxels; FOV = 256 mm).

#### Processing and ROI Delineation

Images were transferred to an SGI/Linux workstation for morphometric analysis. Cortical reconstruction was performed using the FreeSurfer image analysis suite^1^. FreeSurfer provides a set of tools to reconstruct topologically correct and geometrically accurate surface models of the inner and outer cortical boundaries, thereby deriving multiple anatomical measures including cortical volume, thickness, and surface area. All FreeSurfer image processing was conducted on a high performance computing facility at the Melbourne Neuropsychiatry Centre, Melbourne, Australia.

The vlPFC ROI was created by combining the pars opercularis, pars triangularis, and pars orbitalis, as labeled by FreeSurfer (Fischl et al., 2004; Desikan et al., 2006). In order to include only prefrontal regions, a coronal cut was applied at Talairach coordinate *y* = 26. This was done to conform to the conservative Talairach criteria described by Rajkowska and Goldman-Rakic (1995). Cortical thickness was measured as the distance between the gray/white matter boundary and the pial surface (i.e., gray/cerebral spinal fluid boundary) at each point on the cortical mantle. FreeSurfer’s automated procedure involves the assignment of a neuroanatomical label to each voxel in an MRI volume based on probabilistic information estimated automatically from a manually labeled training set. All MRI data checking was completed by a researcher blinded to participant characteristics. All images were checked to ensure that FreeSurfer’s automatic preprocessing stream was accurate. As FreeSurfer requires removal of the dura and skull in order to estimate cortical thickness, accurately, all images where dura and/or skull remained were manually edited by the first author. Three participants were subsequently excluded based on poor quality images. The data for each participant were resampled to an average participant and surface smoothing was performed using the qcache command and a 10-mm full-width half-maximum Gaussian kernel before statistical analysis.

### Data Analysis

Pearson’s bivariate correlations were performed to assess associations between all measures. Regression analyses were performed to test the moderating relationship of the three cortisol measures (CAR, diurnal slope, and AUC_ground_), and sex, on the association between self-reported victimization and vlPFC volume, surface area, and cortical thickness. All regression analyses were performed in MPlus, and full information maximum likelihood (FIML) was used to account for missing data. All regression analyses were tested against a false discovery rate (FDR; Narum, 2006) corrected alpha of 0.02727 in order to correct for the three regression analyses performed per cortisol measure. Subsequent simple slopes analyses (based on listwise data) were used to investigate the nature of significant moderator effects.

## Results

### Descriptive Statistics

A final sample of 50 (24 boys) participants was used in the analyses. These participants represented those with non-missing dependent variables (i.e., useable MRI data). Of these 50, CAR and diurnal slope was available for 41 children; AUC_ground_ was available for 40 children due to missing data for one child at one of the five time points. Victimization data was available for 41 participants. The raw cortisol data were log-transformed to normalize skewness. All analyses were performed using the transformed variables. Table 1 shows the descriptive data for all study variables. Right vlPFC volume differed by sex (dummy coded; girls = 1, boys = 0), with boys having larger volume than girls. Boys and girls did not differ on victimization, bilateral vlPFC thickness, surface area, left hemisphere volume, CAR, diurnal slope, and AUC_ground_ (see Table 2 for details). Victimization was uncorrelated with all three cortisol measures (*p* > 0.05). Similarly, no significant correlations were found between victimization and vlPFC structure, or between cortisol and vlPFC structure (*p* > 0.05). The correlations between variables are presented in Table 3.

**Table 1:** Descriptive statistics for all study variables.

	*N*	*M*	*SD*	Range
1. Sex	50 (24 boys)	0.52	0.50	0–1
2. Victimization	41 (20 boys)	7.95	3.04	4–16 [4–20]
3. CAR	41 (19 boys)	0.18 nmol/L	0.23	−0.35–0.76
4. Diurnal slope	41 (19 boys)	1.15 nmol/L	0.27	0.46–1.63
5. AUC_ground_	40 (19 boys)	496.03 nmol/L	136.93	238.21–1049.95
6. lh vlPFC surface area	50	1875.80 mm^2^	327.41	1386–2855
7. rh vlPFC surface area	50	1987.78 mm^2^	293.56	1509–2984
8. lh vlPFC thickness	50	2.81 mm	3.07	2.42–3.07
9. rh vlPFC thickness	50	2.82 mm	1.34	2.48–3.04
10. lh vlPFC volume	50	6109 mm^3^	1234.22	3900–9785
11. rh vlPFC volume	50	7029.58 mm^3^	1148.81	5035–10610

*CAR, cortisol awakening response; AUC_ground_, area under the curve with respect to ground; vlPFC, ventrolateral prefrontal cortex. lh denotes left hemisphere and rh denotes right hemisphere. Square brackets denote possible range. Sex dummy coded (1, girls and 0, boys).*

**Table 2:** *T*-tests for victimization, AUC_ground_, CAR, slope, and vlPFC surface area, thickness, and volume by sex.

	*t*	df	*p*
Victimization	0.61	39	0.546
CAR	−0.28	39	0.780
Slope	−0.06	39	0.954
AUC_ground_	0.20	38	0.840
lh vlPFC surface area	1.86	40.87	0.071
rh vlPFC surface area	1.93	48	0.059
lh vlPFC thickness	0.59	48	0.560
rh vlPFC thickness	1.97	48	0.055
lh vlPFC volume	1.76	48	0.086
rh vlPFC volume	2.06	48	0.045^∗^

*CAR, cortisol awakening response; AUC_ground_, area under the curve with respect to ground; vlPFC, ventrolateral prefrontal cortex. lh denotes left hemisphere and rh denotes right hemisphere. Sex dummy coded (1, girls and 0, boys); ^∗^p < 0.05.*

**Table 3:** Pearson correlations of predictor and outcome variables.

	1	2	3	4	5	6	7	8	9	10	11
1. Sex	–										
2. Victimization	−0.10	–									
3. CAR	0.05	0.09	–								
4. Diurnal slope	0.01	0.03	0.39^∗^	–							
5. AUC_ground_	−0.03	−0.14	−0.10	−0.36^∗^	–						
6. lh vlPFC surface area	−0.26	−0.10	0.08	0.14	−0.06	–					
7. rh vlPFC surface area	−0.27	−0.06	−0.06	0.14	−0.10	0.98^∗∗^	–				
8. lh vlPFC thickness	−0.08	−0.10	0.07	0.30	−0.21	0.27	0.27	–			
9. rh vlPFC thickness	−0.27	−0.08	−0.02	0.23	−0.01	0.16	0.13	0.68^∗∗^	–		
10. lh vlPFC volume	−0.25	−0.13	−0.07	0.15	−0.11	0.94^∗∗^	0.92^∗∗^	0.53^∗∗^	0.33^∗∗^	–	
11. rh vlPFC volume	−0.29^∗^	−0.10	−0.08	0.16	−0.10	0.94^∗∗^	0.95^∗∗^	0.42^∗∗^	0.40^∗∗^	0.94^∗∗^	–

*CAR, cortisol awakening response; AUC_ground_, area under the curve with respect to ground; vlPFC, ventrolateral prefrontal cortex. lh denotes left hemisphere and rh denotes right hemisphere. Sex dummy coded (1, girls and 0, boys); ^∗^p < 0.05; ^∗∗^p < 0.01.*

### Moderating Role of CAR

No significant relationships were found pertaining to vlPFC surface area, thickness, or volume (all *p’s* > 0.02727).

### Moderating Role of Diurnal Slope

No significant relationships (main effects or interactions) were found pertaining to vlPFC surface area, volume, or thickness (all *p’s* > 0.02727). The second order interaction (Victimization × Diurnal slope × Sex) was found to predict right hemisphere vlPFC surface area (β = 1.321, *SE* = 0.568, *p* = 0.027). This result remained significant when also controlling for intracranial volume (ICV) at an uncorrected alpha level (β = 0.365, *SE* = 0.176, *p* = 0.038). Regression analyses performed for boys and girls separately for the significant interaction revealed that the interaction between victimization and diurnal slope predicted right hemisphere vlPFC area for boys (β = −0.957, *SE* = 0.326, *p* = 0.003), but not girls (*p* > 0.6).

#### Simple Slopes Analysis

**FIGURE 1:**
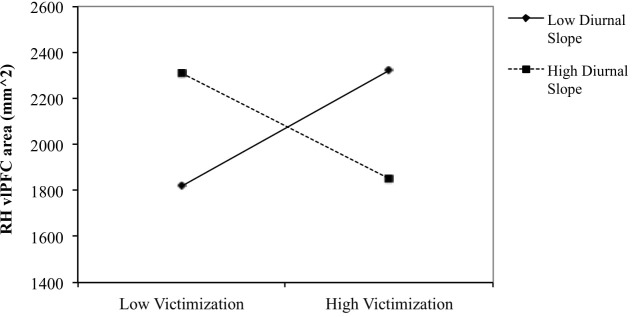
Right vlPFC surface area in relation to victimization for flatter (–1 SD) and steeper (+1 SD) diurnal slope in boys. The slope was negative for boys with steeper diurnal slope [gradient = –85.57, *t*(24) = –2.069, *p* = 0.052] and positive for boys with flatter diurnal slope [gradient = 87.46, *t*(24) = 1.834, *p* = 0.082].

To examine the nature of the interaction effect for boys of peer victimization and diurnal slope on right hemisphere vlPFC area, the relationship between peer victimization and vlPFC area was determined for boys scoring low (1 SD below the mean) and high (1 SD above the mean) on diurnal slope (see Figure 1). The simple slope was negative for boys with steeper diurnal slope [*gradient* = −85.57, *t*(24) = −2.069, *p* = 0.052], and positive for boys with flatter diurnal slope [*gradient* = 87.46, *t*(24) = 1.834, *p* = 0.082].

### Moderating Role of AUC_ground_

No significant relationships were found pertaining to bilateral vlPFC volume or thickness. The second order interaction (Victimization × AUC_ground_ × Sex) was found to predict right hemisphere surface area (β = −0.406, *SE* = 0.165, *p* = 0.019). This result remained significant when also controlling for intracranial volume (ICV) at an uncorrected alpha level (β = −0.111, *SE* = 0.052, *p* = 0.032). Regression analyses performed for boys and girls separately for the significant interaction revealed that the interaction between victimization and AUC_ground_ predicted right hemisphere vlPFC area for boys (β = 0.367, SE = 0.154, *p* = 0.025), but not girls (*p* > 0.5).

#### Simple Slopes Analysis

**FIGURE 2:**
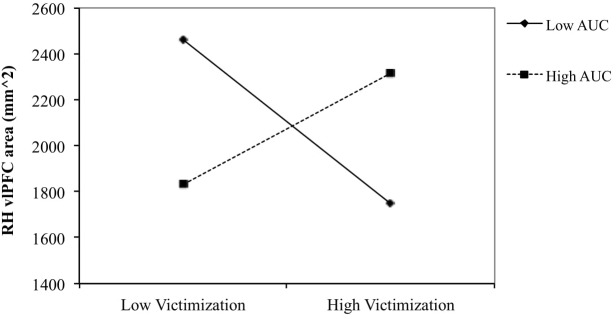
Right vlPFC thickness and surface area in relation to victimization for low (–1 SD) and high (+1 SD) AUCground in boys. The slope was negative for boys with low AUCground [gradient = –127.14, *t*(24) = –1.774, *p* = 0.091] and positive for boys with high AUCground (*p* = 0.167).

To examine the nature of the interaction effect for boys of peer victimization and AUC_ground_ on right hemisphere vlPFC area, the relationship between peer victimization and vlPFC area was determined for boys scoring low (1 SD below the mean) and high (1 SD above the mean) on AUC (see Figure 2). The simple slope was negative for boys with low AUC_ground_ [*gradiant* = −127.14, *t*(24) = −1.774, *p* = 0.091], and non-significant for boys with high AUC_ground_ (*p* = 0.167).

## Discussion

This study addressed the relationship between childhood peer victimization, cortisol, and adolescent vlPFC structure. To our knowledge, this is the first study assessing the effect of HPA-axis function on the relationship between victimization and adolescent brain structure. Consistent with expectations, we found that cortisol moderated the relationship between childhood victimization and adolescent vlPFC structure in a sex-dependent manner. Specifically, we showed that high childhood victimization in boys with high versus low cortisol levels was differentially related to vlPFC structure in adolescent boys.

Although not the primary aim of the study, we also tested for direct associations between victimization and cortisol measures. There were no significant associations. Earlier studies examining the relationship between victimization and cortisol have often found flatter CAR, lower overall cortisol secretion, and lower cortisol reactivity in victimized children (Knack et al., 2011; Ouellet-Morin et al., 2011). However, several studies fail to report a relationship between concurrent cortisol and victimization (Peters et al., 2011; Vaillancourt et al., 2011). These inconsistencies may be due to differences in methodologies used, but also point to the fact that victimized individuals may differ in their HPA-axis function.

We found that, for boys, self-reported victimization was differentially related to vlPFC structure depending on cortisol levels. The higher boys scored on childhood peer victimization and the higher their AUC_ground_, the larger their right hemisphere vlPFC surface area was as an adolescent. A different influence of cortisol on the relationship between victimization and vlPFC structure was observed when considering the diurnal slope. Boys who scored higher on victimization and who had flatter diurnal slope had a larger right hemisphere vlPFC surface area as adolescents. These results are consistent with our hypothesis that cortisol moderates the relationship between victimization and brain structure. This finding may indicate a biological sensitivity to stress that could influence structural brain development.

Although our moderation effects were consistent with hypotheses, the directions of associations were not. We hypothesized that victimization would be associated with smaller vlPFC in adolescents with higher cortisol output. Instead, we found that victimization was associated with smaller vlPFC area in individuals with lower AUC and steeper slope. While we did not assess structural changes in the brain over time, the smaller vlPFC surface area in victimized children with lower daily cortisol output may indicate normal or precocious adolescent brain development. In normally developing children, surface area initially shows expansion until the age of about 12.7 years, after which steady reductions are observed (Schnack et al., 2014). Moreover, right hemisphere surface area decreases at a faster rate than the left (Schnack et al., 2014). Reductions in surface area may indicate increased specificity, increased neuronal efficiency, and temporal precision (Lewis, 1997; Rutherford et al., 1998; Bullmore and Sporns, 2010). Prefrontal cortex activity in particular becomes more focal during adolescence (Vijayakumar et al., 2014) and increased cortical thinning and reduced surface area has been associated with higher cognitive ability in mid-adolescence (Tamnes et al., 2013; Schnack et al., 2014). Conversely, less cortical surface area reduction in adolescence has been associated with conduct disorder symptoms (Sarkar et al., 2015). Given the support for cortical surface area reduction in adolescence as part of normal development, it is plausible that high cortisol represents an adaptive response to a stressor, which may be protective.

The length of time that a stressor has been present can impact diurnal cortisol levels (Miller et al., 2007). It has been suggested that stressors that have been present for a short time are linked to having elevated cortisol, whereas long-term stress has been associated with flattened cortisol slopes (Fries et al., 2005). It is possible that children who have only been bullied for a short while are still more biologically reactive to stress compared to children who have been bullied over a longer period of time (Knack et al., 2011). While higher cortisol levels may indicate a flexible stress response that is able to cope with a current stressor, it does indicate that the organism is experiencing stress. As such, we cannot exclude the possibility that this may lead to deleterious outcomes through a mechanism not assessed here. For example, high cortisol has been associated with a range of negative outcomes including depression, diabetes mellitus, and chronic immune disorders (McEwan, 1998). These findings echo Miller et al. (2007) by suggesting that the consequences of an up- or down-regulated stress system depend largely on characteristics of the stressor and the organism. This emphasizes the need for adequate characterization of the stressor and for taking potential confounders into account.

As our findings indicated significant sex differences, it is important to consider them in the light of earlier findings relating to sex differences. While sex differences in cortical development across adolescence are unclear, females’ cortical volume and surface area has been found to “peak” earlier than males’ (Raznahan et al., 2010). Wierenga et al. (2014) found similar effects for volume and thickness development within some prefrontal regions. It is possible that the sex differences observed in our study relate to pubertal changes. Future studies should take into account pubertal status and measures of gonadal hormones and their complex interplay with HPA-axis activity (Simmons et al., 2015).

This study has some limitations. Victimization is only one aspect of children’s experiences and does not exist in isolation. Future studies should consider other factors that may increase vulnerability to or confer protection against the impact of peer victimization. Given that measures were not repeated across development, it is not possible to infer how victimization might influence HPA-axis function and brain development over time. Future studies would do well to assess vlPFC structure at several time points so as to assess the trajectory of brain development, and also perform a whole-brain analysis to investigate possible effects on other brain regions. With a relatively small sample size (*n* = 50, 24 boys), these results should be generalized with caution and be replicated with a larger sample. Also due to the small sample, we were unable to consider various subtypes of bullying. This may be problematic as different types of victimization have been associated with distinct behavioral outcomes (Vaillancourt et al., 2008). Despite the relatively small number of participants, the study was conducted with a population-based sample, which supports the external validity of our findings. The rate of victimization found in our sample was similar to that of earlier studies (Vaillancourt et al., 2011), though it should be noted that our sample contained few children who considered themselves to be highly victimized. This could indicate that the Olweus Bully/Victim scale may not be sensitive enough to capture the full extent of victimization in our sample. The questionnaire asked children to report on their victimization experiences in the past year and children may not have been able to report this accurately. This would have had direct implications for the validity of our cortisol measures given that the relationship between stress and cortisol levels changes over time (Miller et al., 2007). Future studies should not only take the frequency of victimization into account, but should also assess the time of victimization onset.

Finally, while cortisol provides helpful insights into biological stress it only represents one aspect of experienced stress. As such, a subjective measure of stress would have been helpful to provide insights into the effect of victimization on the developing brain. Recently it was shown, for example, that peer victimization is related to greater pro-inflammatory cytokine responses to an acute social stressor in adolescents (Giletta et al., 2018). Future studies should combine both subjective and biological measures of stress and assess these in relation to victimization and brain structure. In addition, diurnal cortisol represents only one aspect of the biological stress response and does not take into account how children may respond when faced with a stressor. Future studies should consider cortisol reactivity to a stressor (victimization or a simulation thereof) to elucidate the relationships between negative peer experiences and biological measures of stress.

## Conclusion

Our study showed that cortisol levels in childhood have a differential effect on the relationship between childhood peer victimization and adolescent vlPFC structure, but only for boys. These findings shed some light on the complex association of cortisol with stress. It is possible that, for boys who are more biologically sensitive to stress (as evidenced by high cortisol), the experience of victimization could have deleterious consequences for brain development. However, questions remain regarding what is desirable in terms of prefrontal brain development during early adolescence. The question of whether low or high cortisol is adaptive in terms of structural brain development should be studied using longitudinal designs assessing stress and brain structure at multiple time points.

## Author Contributions

BG, SS, and AC contributed to the conception or design of the work and the acquisition of data. MdP and SW contributed to the analysis of the data. MdP, SW, and BG contributed to the interpretation of the data. MdP drafted the work. All authors revised the manuscript critically for important intellectual content and provided approval for publication of the content.

## Conflict of Interest Statement

The authors declare that the research was conducted in the absence of any commercial or financial relationships that could be construed as a potential conflict of interest. The reviewer AT and handling Editor declared their shared affiliation.

## Footnotes

1 http://surfer.nmr.mgh.harvard.edu/
